# Polymer-based chromophore–catalyst assemblies for solar energy conversion

**DOI:** 10.1186/s40580-017-0132-z

**Published:** 2017-12-22

**Authors:** Gyu Leem, Benjamin D. Sherman, Kirk S. Schanze

**Affiliations:** 1 Department of Chemistry, University of Texas at San Antonio, San Antonio, TX 78249 USA; 2 Department of Chemistry & Biochemistry, Texas Christian University, Fort Worth, TX 76129 USA

**Keywords:** Energy conversion and storage, Energy and charge transport, Ru-containing polymer system, Dye-sensitized photoelectrochemical cells, Water oxidation, Photoanode, polymeric chromophore-water oxidation

## Abstract

The synthesis of polymer-based assemblies for light harvesting has been motivated by the multi-chromophore antennas that play a role in natural photosynthesis for the potential use in solar conversion technologies. This review describes a general strategy for using polymer-based chromophore–catalyst assemblies for solar-driven water oxidation at a photoanode in a dye-sensitized photoelectrochemical cell (DSPEC). This report begins with a summary of the synthetic methods and fundamental photophysical studies of light harvesting polychormophores in solution which show these materials can transport excited state energy to an acceptor where charge-separation can occur. In addition, studies describing light harvesting polychromophores containing an anchoring moiety (ionic carboxylate) for covalent bounding to wide band gap mesoporous semiconductor surfaces are summarized to understand the photophysical mechanisms of directional energy flow at the interface. Finally, the performance of polychromophore/catalyst assembly-based photoanodes capable of light-driven water splitting to oxygen and hydrogen in a DSPEC are summarized.

## Introduction

Artificial photosynthesis mimics the natural process occurring in plants with specifically engineered light-harvesting systems to carry out the direct transformation of light energy to that stored in a chemical bond (i.e. a solar fuel) [1]. The ultimate goal in artificial photosynthesis is to generate carbon-based fuels from water splitting and CO_2_ reduction with sunlight as shown in Eq 1 [2].

1 $$2{\text{H}}_{ 2} {\text{O}} + {\text{CO}}_{2} + 8{\text{ h}}\upnu \to {\text{CH}}_{4} + 2{\text{O}}_{2} \;\Delta {\text{G}}^\circ = 10.3\;{\text{eV}} .$$

**Fig. 1:**
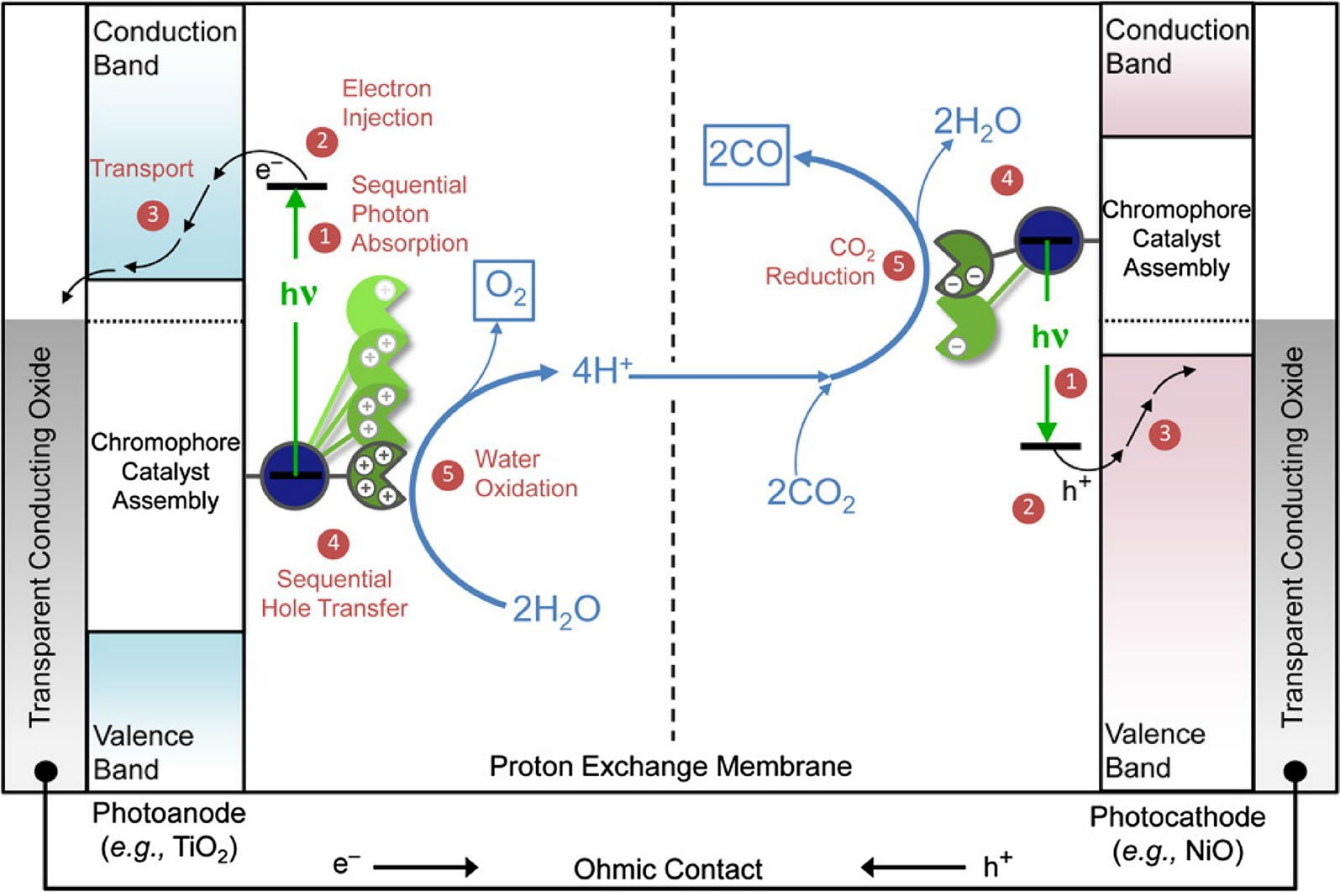
Schematic diagram for a tandem DSPEC for solar-driven CO_2_ splitting into CO and O_2_ by the net reaction, 2CO_2_ + 4 hν → 2CO + O_2_ (Reproduced with permission from Ref. [5]. Copyright 2016 American Chemical Society)

Toward this goal, artificial photosynthesis as an integrated system requires multifunctional behavior that generally consist of three parts: collection of light across a broad portion of the solar spectrum, transportation of photo excited-energy to charge separation sites of pigment (or chromophore) assemblies, and utilization of the photoexcited carriers to drive catalytic reactions that produce chemical fuel [3, 4]. Integration of multiple components to facilitate these photoelectrochemical events is necessary to achieve highly efficient solar-to-fuel conversion. In order to develop artificial photosynthesis, dye-sensitized photoelectrochemical cells (DSPECs) have been designed and investigated to provide an architecture to drive water splitting and CO_2_ reduction at n- and p-type semiconductor oxides with molecular-level light absorption and catalysis as shown in Fig. 1 [5, 6]. Surface-bound chromophore–catalyst assemblies at the photoanode and photocathode absorb light and perform the solar fuel half-reactions. To date DSPEC systems have integrated light harvesting assemblies utilizing a variety of architectures such as polymers, dendrimers, and peptides [7–13].

In natural photosynthesis, a multi-chromophore antenna system absorbs light efficiently and transmits excited-state energy rapidly to a reaction center. Related antenna strategies can be achieved with polychromophoric polymers. Ruthenium(II) polypyridyl complexes have been widely used over the last decades as light harvesting chromophores due to their high absorptivity in the visible spectrum for a strong metal-to-ligand charge transfer (MLCT) transition [11, 14–17]. Ruthenium(II) polypyridine complexes incorporated into a polymer scaffold offer a potential means of developing light-harvesting antenna systems for applications in dye-sensitized photovoltaic cells and artificial photosynthesis. According to previous studies, MLCT excitons in polymeric chromophore systems exhibit a site-to-site hoping mechanism along the polymer chains [18, 19]. In the past several decades there have been a number of reported polymeric chromophore systems containing pendant ruthenium(II) polypyridine as light harvesting units because of beneficial properties such as remarkable photo- and thermal stability and long-distance exciton and charge transport [20–25]. Recently, our research group has carried out investigations of the photophysical and electrochemical properties of novel polychromophores or polychromophore–catalyst assemblies in order to understand the photodynamics of charge and exciton transport in solution and at the interface of semiconductor materials [19, 26, 27].

Constructing films of defined molecular composition has been a priority of applied material and interface science. Layer-by-layer (LbL) polyelectrolyte self-assembly offers a simple and versatile tool to allow facile control of molecular assemblies prepared directly on substrate films [28]. Most LbL polyelectrolyte films feature multilayers composed of positively and negatively charged polyelectrolytes and employ the electrostatic Coulomb interaction between the oppositely charged macromolecules to form stable films. LbL technology has promising applications in solid-state light-emitting devices [29], drug delivery [30], biomembrane [31], electrodialysis membranes [32], and diagnostics [33]. Very recently, Leem et al. used the LbL approach to construct multilayers for the use in a DSPEC [26].

This review is focused on the synthesis, and photophysical properties of polymeric assemblies consisting of multiple Ru(II) polypyridyl complexes as well as the polyelectrolyte LbL chromophore–catalyst assembly deposited onto semiconductor substrates for use in a DSPEC. The review highlights light-driven water oxidation using a polymeric chromophore–catalyst assembly immobilized onto a semiconductor at a DSPEC photoanode.

## Polymer-based metal complex assemblies

### Synthesis and characterization of polymer-based metal complex assemblies

Our research group has been interested in developing polymerization strategies to prepare functionalized polystyrenes that feature pendant metal–organic chromophores and to investigate the photophysical and electrochemical properties of the chromophoric sites preserved in the polymer. As an early synthetic strategy, polymer assemblies containing a [tris(bipyridyl)ruthenium(II) derivatives] were considered for creating polymer assemblies that could be modified by introducing polypyridyl Ru complexes [24, 25, 34].

Polypyridyl complexes of ruthenium were attached to styrene-*p*-(chloromethyl) styrene copolymers derivatized via nucleophilic displacement of chloride followed by the formation of an ether (**1**) or ester linkage (**2**) in Table 1 [24]. Based on electronic absorption spectral data of ether and amide-linked polymers, the photophysical properties of the metal to ligand charge-transfer transitions of ruthenium(II) complexes on the polymeric chain are unchanged upon binding to the polymers. For an amide-linked polymer (**3**), the aminated poly(styrene-*p*-(aminomethyl)styrene) (*co*-PS-CH_2_NH_2_) can be coupled with a carboxylic acid derivative of the Ru metal complex in the presence of a couple of reagents [25, 34]. The amide-linked polymer strategy is a preferable coupling chemistry because of the availability of a variety of carboxylic acid derivatives of metal chromophores and quenchers. More interestingly, intra-strand energy transfer in the amide-linked polymer is known to be rapid compared to energy transfer in the ether-linked polymer due to the electron withdrawing effect of the amide link relative to the ether link.

**Table 1:** Monofunctional polymers containing Ru(II) chromophoric sites

Polymer						
Abs_max_^a^ dπ − π* (nm)	454 (1) 478 (2)	458	458	451	460	456
λ_em_ (nm)	655 (1) 658 (2)	646	645	623	640	657
Refs	[24]	[25, 34]	[35]	[22]	[21]	[23]

^a^Absorption maximum (± 3 nm) for the most intense metal-to-ligand charge transfer (MLCT) band measured in acetonitrile solution

The polymer poly(4(2-[*N*,*N*-bis(trimethylsilyl)amino]ethyl)styrene) (**4**) was prepared by a living anionic polymerization method that offers well-controlled polymer chain lengths and narrow polydispersity (M_w_/M_n_ < 1.2) compared to the previous polymer (**3**) that was prepared by free radical polymerization and had a PDI of 1.5 [35]. The amide coupling reaction was carried out to link a ruthenium polypyridyl complex to the amino polymer. A major drawback of this anionic polymerization is monomer functional group tolerance under the strongly basic reaction conditions. As a key advantage, however, that aids the investigations of the photophysical properties of the polymer, the amino polymers synthesized by this method are linear and optically transparent in the visible region.

Our group reported the synthesis of a polystyrene backbone by the reversible addition-fragmentation chain transfer (RAFT) polymerization method combined with the azide-alkyne Huisgen cycloaddition as a “click chemistry” reaction [22]. The azide-alkyne click reaction affords a high yield for grafting Ru(II) complexes containing a terminal acetylene group onto the methyl azide functionalized polystyrene backbone. This is followed by the formation of a triazole linker under mild reaction conditions at room temperature. From emission quantum yield and lifetime studies, the MLCT excited state is efficiently quenched in the RAFT PS-Ru polychromophores (**5**) relative to a model Ru complex chromophore in the absence of a polymer backbone. Interestingly, the thiol (–SH) end-groups on the polymers from the RAFT chain transfer agent can quench the MLCT state by a charge-transfer mechanism among the chromophore, leading to considerable reduction in the lifetime of the MLCT state.

Recently, in order to eliminate the thiol polymer end-groups we developed atom transfer radical polymerization (ATRP) and nitroxide-mediated controlled radical polymerization (NMP) methods to prepare polystyrene-based polychromophores with pendant Ru(II) polypyridyl complexes [18, 20, 21, 23]. The ATRP method allows for postpolymerization modification by incorporating –Br end group functionality [21]. The polypyridylruthenium derivatized polystyrenes were achieved in two steps. The polystyrene backbone was prepared by ATRP of the *N*-hydroxyscuccinimide (NHS) derivative of 4-vinyl benzoate with methyl α-bromoisobutyrate as the initiator. Subsequently an amide coupling reaction of the NHS-polystyrene with Ru(II) complexes derivatized with aminomethyl groups ([Ru(bpy)_2_(CH3-bpy-CH_2_NH_2_)]^2+^) afforded polypyridylruthenium derivatized polystyrenes, (**6**).

**Fig. 2:**
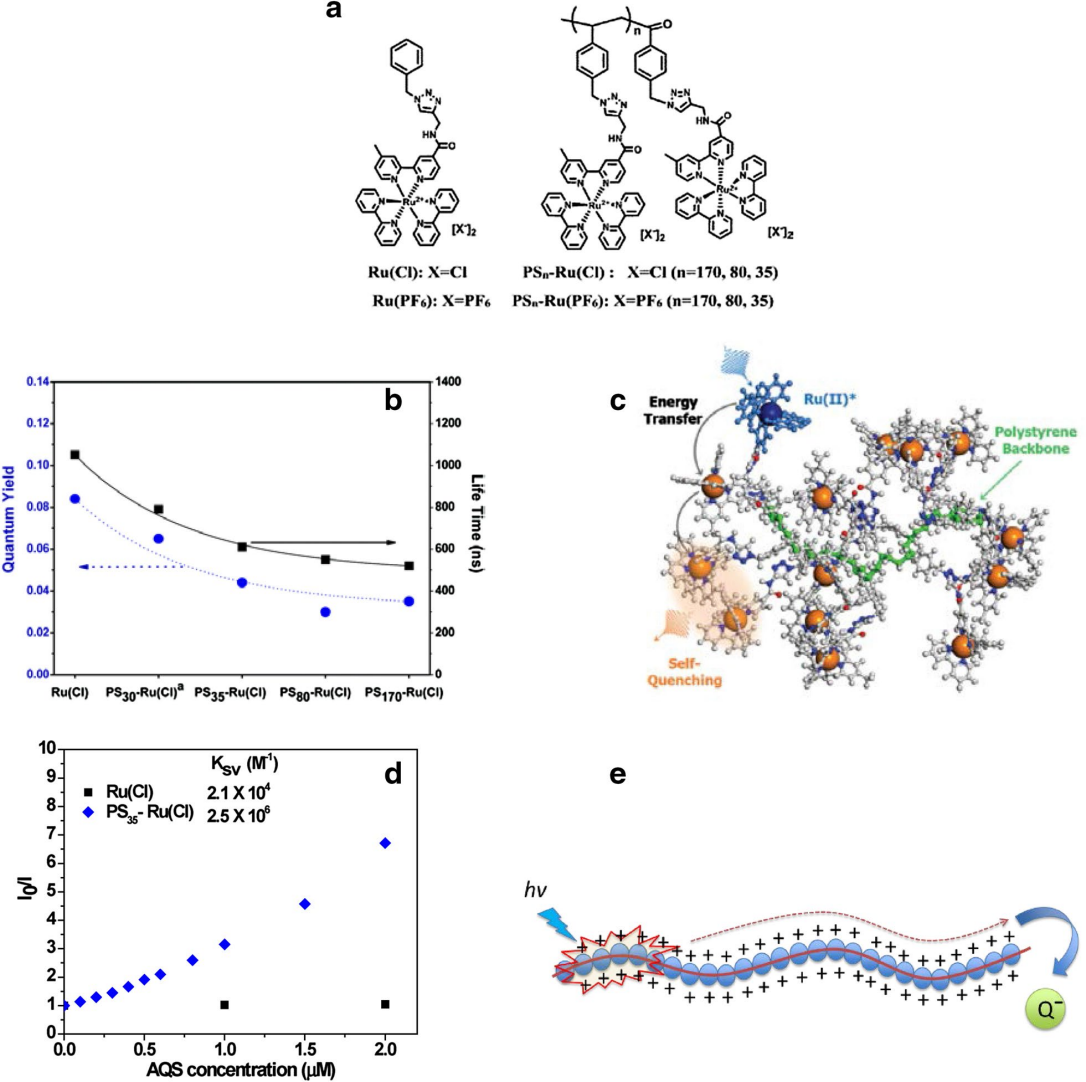
**a** Structures of the model complex, Ru(PF_6_) and Ru(Cl) and ruthenium derivatized polystyrene (PS_n_-Ru(PF_6_) and PS_n_-Ru(Cl)). **b** Plot of the lifetime (black square) and quantum yield (blue circle). **c** Proposed energy transfer and self-quenching mechanisms. **d** Stern–Volmer plot (I_0_/I vs. [AQS]) for emission quenching of Ru(Cl), and PS_35_-Ru(Cl) with AQS by monitoring the emission intensity at 673 nm. **e** Illustration of excition quenching in the presence of quencher (Reproduced with permission from ref [23]. Copyright 2015 the Royal Society of Chemistry)

Leem et al. [23] explored systematic structure–property relationships using a series of polystyrene backbone polymers containing Ru(II) polypyridyl pendant chromophores, (**7**). The NMP and “click chemistry” methodology affords polymers with precisely defined number average molecular weights (M_n_) ranging from ~ 5500 to 24,000  g mol^−1^ with a high efficiency for the click grafting of Ru chromophores to the polymer backbone. The emission quantum yield and lifetimes for the polymers in solution systematically decrease with increasing molecular weight as shown in Fig. 2. With an increased loading of Ru metal chromophores on the polymer assemblies, the probability of exciton migration from the MLCT excited states to trap sites within the close-packed Ru pendants in a polymer backbone becomes more pronounced. Exciton-exciton annihlilation also becomes important at high light flux. Importantly, we confirmed the existence of exciton hopping along the polymer chains according to emission quenching studies of the model compound, Ru(Cl) and polymers, PS_n_-Ru(Cl). The luminescence of the cationic Ru(Cl) and PS_n_-Ru(Cl) is quenched by oppositely charged sodium 9,10-anthraquinone-2,6-disulfonate (AQS). Quenching of the polymers exhibited significant amplification (~ 100-fold) in comparison to the quenching of the Ru(Cl).

### Polymeric chromophores on metal oxide films

**Fig. 3:**
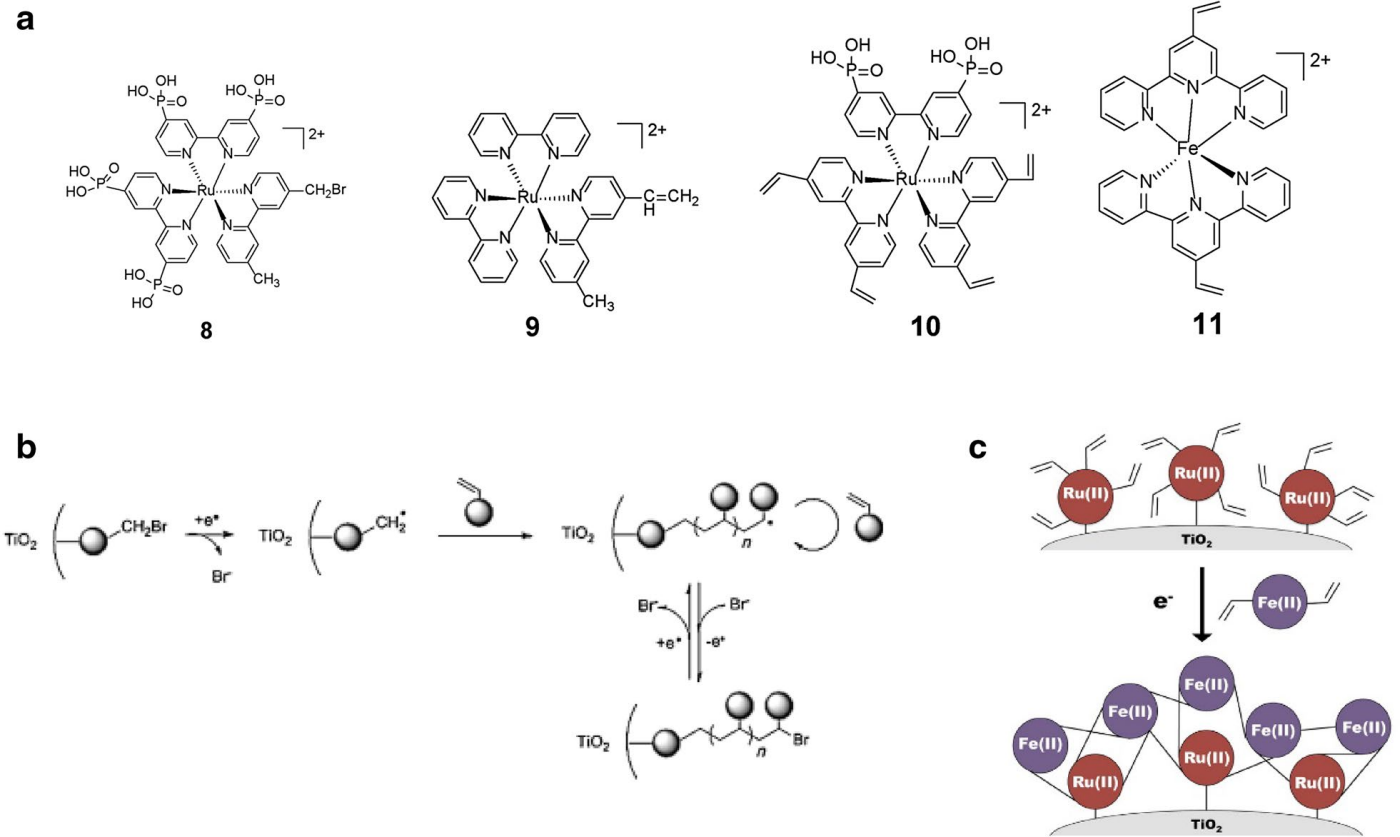
**a** Structures of [Ru(4,4′-PO_3_H_2_-2,2′-bpy)_2_(4-Br-4′-Mebpy)]^2+^ (**8**), [Ru(bpy)_2_(4-Me-4′-vinlybpy)]^2+^ (**9**), Ru(5,5′-(R)2-bpy)_2_Cl_2_ (R=CH=CH_2_) (**10**), [Fe(v-tpy)_2_]^2+^ (v-tpy = 4′-vinyl-2,2′:6′,2″-terpyridine) (**11**). **b** Mechanism of the oligomeric growth of Ru^II^ complexes from **8** in the cavities of the mesoporous TiO_2_ film. **c** Schematic diagram of the surface structure following reductive polymerization of **11** on TiO_2_-**10** (Reproduced with permission from Ref. [36, 37]. Copyright 2014, Wiley–VCH Verlag Gmbh & Co. and 2013, American Chemical Society, respectively)

The Schanze and Meyer research groups have focused on the development of functional multi-chromophore light harvesting assemblies by coupling polymer assemblies to mesoporous semiconductor surfaces for understanding of the structure and dynamical phenomena relating to solar energy conversion systems. For example, covalently or noncovalently surface-bound polychromophoric assemblies have been used that contain anchoring group (i.e. –COOH and PO_3_H_2_) or oppositely charged chains to bind the assembly onto a mesoporous semiconductor surface for excited state electron injection into conduction band of semiconductor. As shown in Fig. 3, the initiator site, [Ru(4,4′-PO_3_H_2_-2,2′-bpy)_2_(4-Br-4′-Mebpy)]^2+^ (bpy = 2,2′-bipyridine, Mebpy = 4,4′-diphosphonic acid-2,2′-bipyridine, **8** was covalently bound to the TiO_2_ surface. Then, the reductive initiation of [Ru(bpy)_2_(4-Me-4′-vinlybpy)]^2+^(4-Me-4′-vinylbpy = 4-vinyl-4′-methyl-2,2′-bipyridine), **9** occurs at surface bound **8** onto TiO_2_ nanoparticles by electrochemically controlled radical polymerization [36]. Similarly, [Ru(5,5′-divinyl-2,2′-bipyridine)_2_(4,4′-(PO_3_H_2_)_2_-bpy)]^2+^ containing a phosphonated bipyridine ligand as anchoring group and vinyl functionalized bipyridine ligands, **10** was selected as the monomer precursor [37]. After the immobilization of **10** as the inner molecular layer in mesoporous TiO_2_ film, [Fe(v-tpy)_2_]^2+^ (v-tpy = 4′-vinyl-2,2′:6′,2″-terpyridine), **11** was reductively electropolymerized onto the existing surface film of **10**, which resulted in enhanced photostability in comparison to the surface-bound Ru complex only. With the use of transient absorption spectroscopy of the polymer-loaded TiO_2_, it was shown that photoexcitation of the surface-bound Ru chromophore is followed by electron injection and Fe^II^ to Ru^III^ electron transfer.

**Fig. 4:**
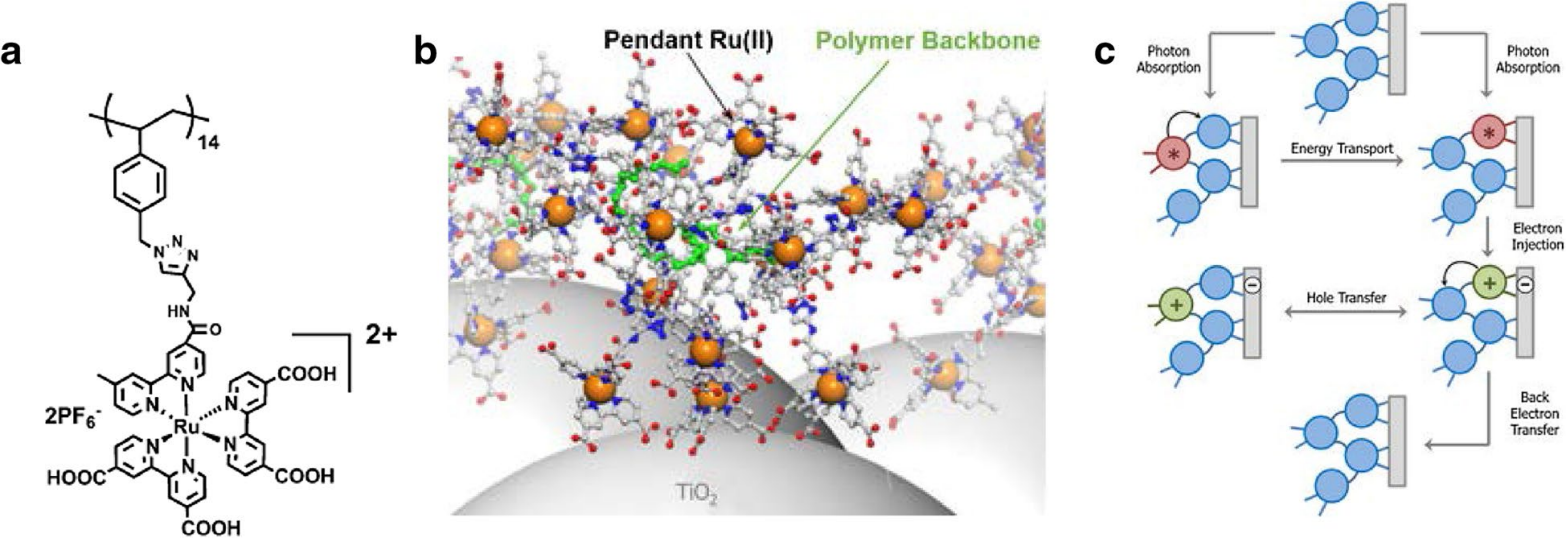
**a** Structure of carboxylic acid-derivatized polymer, **PS**-**Ru**-**A**. **b** Cartoon of PS-Ru-A anchored to a TiO_2_ nanoparticle surface. **c** Schematic representation of the photophysical events of the PS-Ru-A assembly at the surface of TiO_2_. The blue circles represent Ru(L)32 + chromophores while the green and red circles correspond to Ru(L)_3_^3+^ and Ru(L)_3_^2+*^ (Reproduced with permission from Ref. [18]. Copyright 2016, Wiley–VCH Verlag Gmbh & Co)

Instead of electropolymerization of Ru(II) polypyridyl chromophore onto a TiO_2_ surface, Leem et al. [19] reported the preparation of conjugated and non-conjugated [18] polymer-based Ru(II) chromophores with carboxylic acid anchoring groups followed by covalent immobilization of the polymeric chromophores to the surface of semiconductor nanoparticles. The carboxylic acid-derivatized ruthenium-functionalized polymer, **PS-Ru-A**, was obtained by a NMP method and click assembly via an azide-alkyne Huisgen cycloaddition reaction allowing the attachment of Ru(II) polypyridyl to the polystyrene backbone as shown in Fig. 4. PS-Ru-A was anchored to mesoporous TiO_2_ films (TiO_2_//**PS**-**Ru**-**A**) in order to understand the interfacial photophysical dynamics of energy and charge transport among tand he pendant complexes along the polymer chain at the interfaces. The MLCT exciton from Ru(II) diffuses efficiently among pendant Ru complexes that are attached along the polymer backbone. Based on transient absorption spectra and time-resolved emission spectra measurements, an antenna effect of the assembly occurs in TiO_2_//**PS**-**Ru**-**A** consisting of the following photophysical events; (1) photoexcitation of surface-bound Ru^II^ chromophores and electron injection into TiO_2_ on the picosecond time scale, (2) multiple Ru* → Ru energy hopping events after photoexcitation of unbound Ru^II^ chromophores followed by electron injection, (3) intra-assembly hole transfer to pendant complexes away from the TiO_2_ surface, and (4) back electron transfer between Ru^III^ and TiO_2_(e−).

## Polymer chromophore–catalyst assemblies for solar energy conversion

**Fig. 5:**
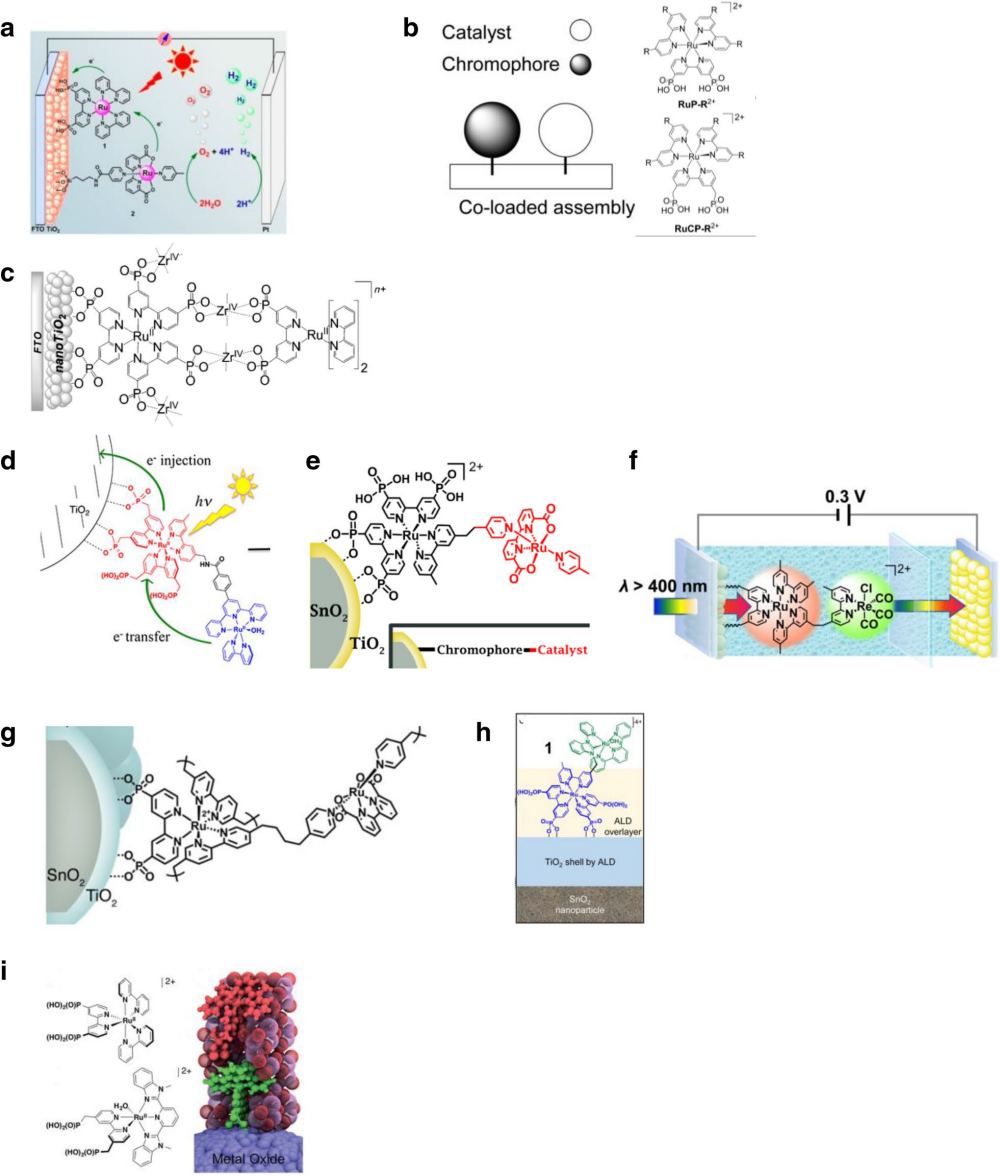
**a**, **b** Co-loaded assembly on TiO_2_ [38, 39]. **c** Bilayer assembly with Zr(IV) bridging on TiO_2_ [40]. (**d**–**f**) Surface formed with pre-synthesized chromophore–catalyst assemblies [41–44]. **g**, **h** Electro-assembly by reductive vinyl coupling [45, 46]. **i** Molecular overlayers [48] (Reproduced with permission from Ref. [38, 39]. Copyright 2012, WILEY‐VCH Verlag GmbH & Co., 2012, 2013, 2015, 2016, and 2017, American Chemical Society, and 2015, The Royal Society of Chemistry)

Multiple strategies have been explored for assembling chromophores and catalysts onto semiconductors including co-loading of chromophore and catalyst species [38, 39], forming bilayer assemblies with Zr(IV)-phosphate bridges [40], adsorbing pre-synthesized covalently linked chromophore–catalyst assemblies [41–44], electro-assembly by reductive vinyl coupling [45, 46], and the deposition of molecular overlayers [47, 48]. An example of a pre-synthesized chromophore–catalyst assembly is shown in Fig. 5, and this approach often requires multistep procedures with poor overall yields. The co-loading strategy requires less synthetic effort with the use of discrete chromophore and catalyst species but with the trade-off of less control of the orientation between the components and less special separation between catalyst and oxide surface. The use of metal cation bridged bilayers addresses the latter shortcoming of co-immobilization by allowing the sequential surface deposition of the phosphate-derivatized chromophore and catalyst species, with the catalyst not in direct contact with the semiconductor surface. While this gives a similar hierarchal surface structure with less synthetic effort as with the use pre-synthesized assemblies, the metal bridge is susceptible to hydrolysis and is generally unstable over long times.

**Fig. 6:**
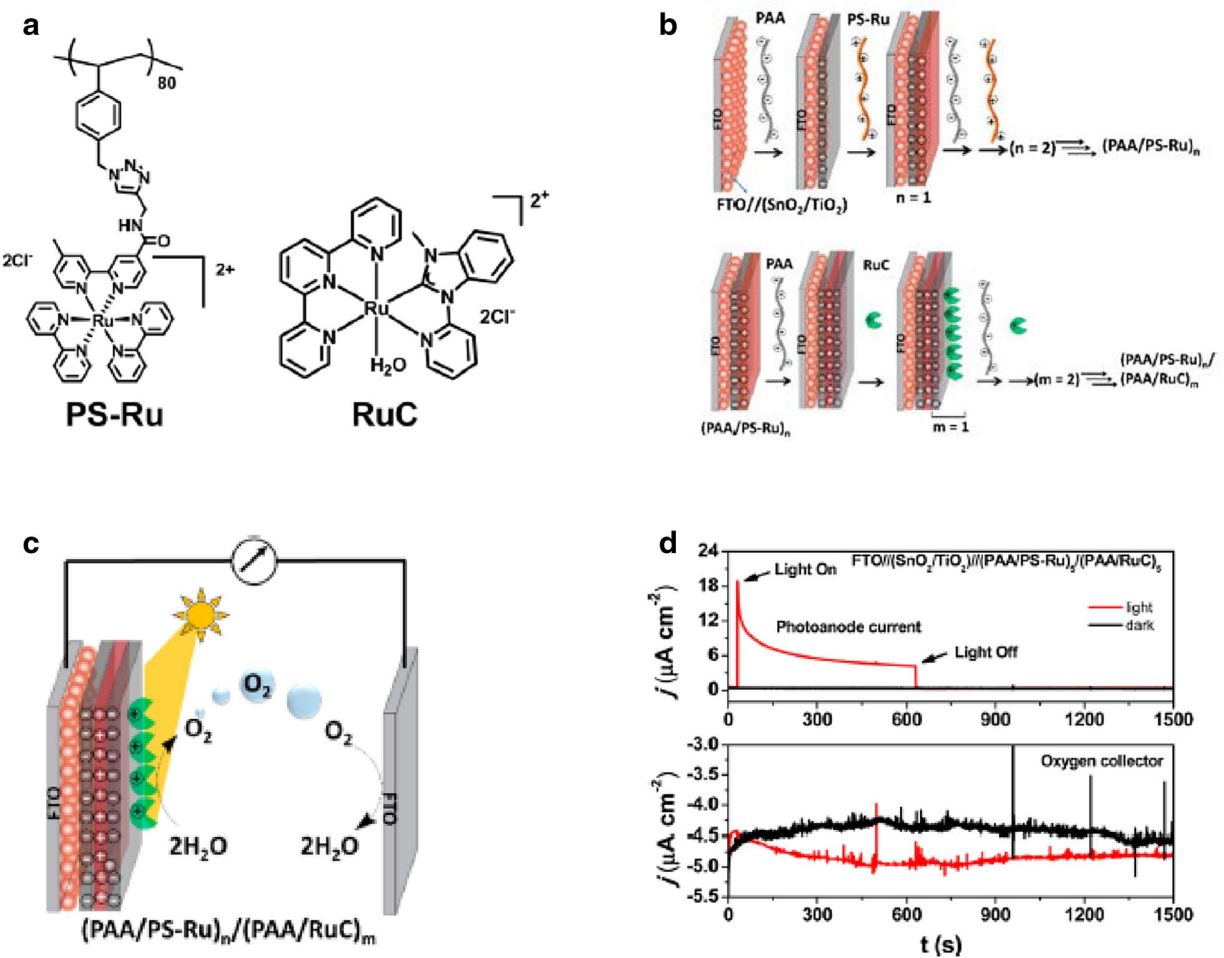
**a** Structures of polychromophore (**PS**-**Ru**) and catalyst (**RuC**). **b** Schematic of the step-wise assembly of the LbL interface using a mesoporous SnO_2_/TiO_2_ core/shell layer deposited on a fluorine doped tin oxide (FTO) substrate. **c** Scheme depicting the C–G setup for detecting photochemically generated O_2_ by the (PAA/PS-Ru)_n_/(PAA/RuC)_m_ prepared electrodes. **d** Current–time traces for the as prepared photoanode under bias with the simultaneous current response measured at the FTO collector electrode shown below (Reproduced with permission from Ref. [26]. Copyright 2016, American Chemical Society)

Our group has been interested in developing a different method for preparing dye-sensitized photoelectrode surfaces referred to as the ‘layer-by-layer’ (LbL) strategy using polychromophore–catalyst assemblies. Figure 6 illustrates the stepwise sequence of preparing an LbL surface with a polystyrene-based Ru polychromophore (**PS**-**Ru**) and the molecular water oxidation catalyst [Ru(tpy)(Mebim-py)(OH_2_)]^2+^ (Mebim-py = 2-pyridyl-*N*-methylbenzimidazole) (**RuC**) [26]. This sequence first involves the deposition of one to several layers of the cationic polymer chromophore chain, with the deposition of an anionic polymer alternated when depositing several thicknesses of the chromophore-containing polymer. The representative study in Fig. 6 details the use of polystyrene-based assemblies featuring pendant [Ru(bpy)_3_]^2+^ chromophores deposited onto mesoporous wide band gap metal oxide substrates, with alternating deposition of poly(acrylic acid) (**PAA**) as an oppositely charged polyelectrolyte via the LbL method. Following the adsorption of the PS-Ru layer, soaking the electrode in solution containing the RuC water oxidation catalyst introduces this species to the surface, with the catalyst layer thickness increased with alternating soakings in a **PAA** solution to build out **RuC**/**PAA** layers on the underlying **PS**-**Ru**/**PAA** surface.

**Fig. 7:**
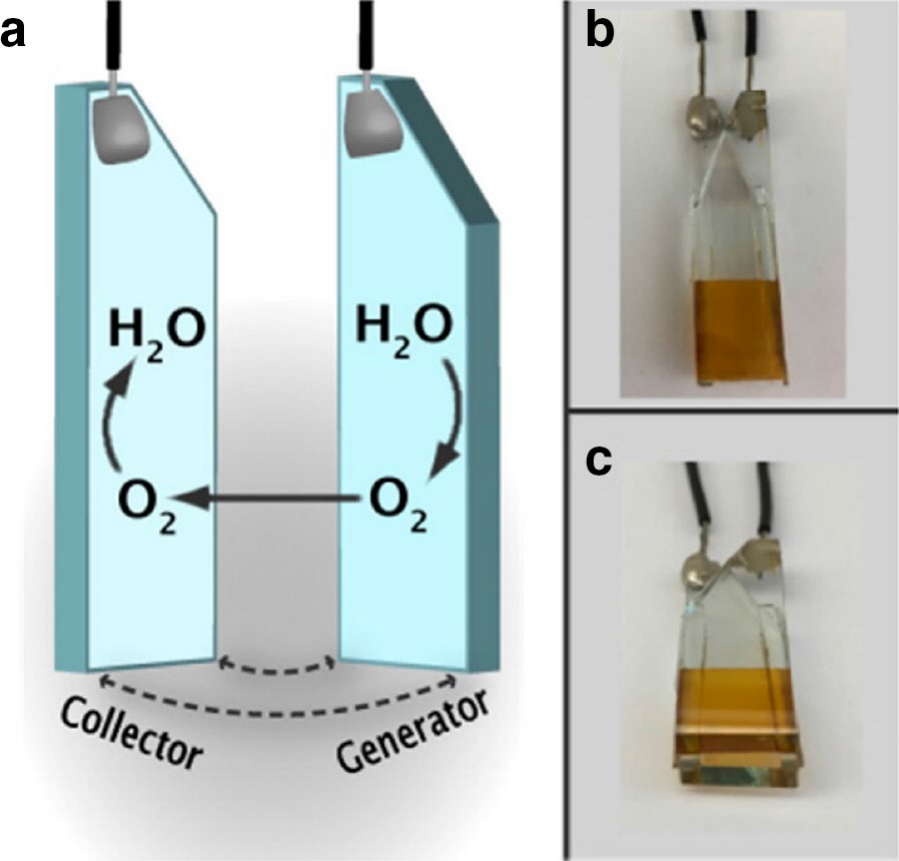
**a** Collector–generator schematic and photographs of the front (**b**) and bottom (**c**) of an example C–G assembly consisting of a FTO|nanoTiO_2_ photoanode derivatized with a chromophore–catalyst assembly and a FTO collector electrode (Reproduced with permission from ref [49]. Copyright 2016 American Chemical Society)

Photoelectrochemical investigations of PS-Ru/RuC were carried out to explore the ability of this surface to support light driven water oxidation in aqueous solution at pH 7 by using a collector-generator (C–G) dual working electrode cell. The C–G electrochemical setup is specifically designed to monitor the in situ photoelectrochemical production of O_2_ by dye-sensitized photoanodes, with the real time response of the setup providing additional information on the photocatalytic stability of the photoanode as shown in Fig. 7 [46, 49, 50]. In addition to the as prepared dye-sensitized photoanode under study (the *generator* electrode), the C–G setup consists of a transparent FTO electrode (the *collector* electrode) held 1 mm from the photoanode surface with the conductive surfaces facing. Narrow, 1 mm thick glass spacers are used to hold the two electrodes at the set distance, and the lateral edges are sealed with inert epoxy to create a small volume between the electrodes. When placed in solution, the volume fills with electrolyte via capillary action and serves to spatially constrain O_2_ produced at the photoanode for accurate detection and quantification at the collector [49].

Using the C–G setup, it was shown that the PS-Ru/RuC LbL system could carry out light driven water oxidation at a SnO_2_/TiO_2_ core/shell electrode with the best performance observed for FTO//(SnO_2_/TiO_2_)//(PAA/PS-Ru)_5_/(PAA/RuC)_5_ [26]. The photocurrent density of this photoanode was ca. 10 μA cm^−2^ after 30 s of illumination (0.44 V vs. NHE bias) and the C–G analysis showed the generation of O_2_ with a 22% Faradaic efficiency. This performance mirrored other DSPEC studies using the same RuC catalyst with more modest overall photocurrent densities than observed compared to the use of [Ru(bda)(L)_2_]-type water oxidation catalysts (bda = 2,2′-bipyridine-6,6′-dicarboxylate; L = neutral donor ligand) [51–53]. The performance of the PS-Ru/RuC LbL system did demonstrate the viability of this approach for to preparing photoelectrode interfaces and improvement will likely occur through further optimization of the LbL deposition strategy and introduction of more active molecular catalysts such as [Ru(bda)(L)_2_].

**Fig. 8:**
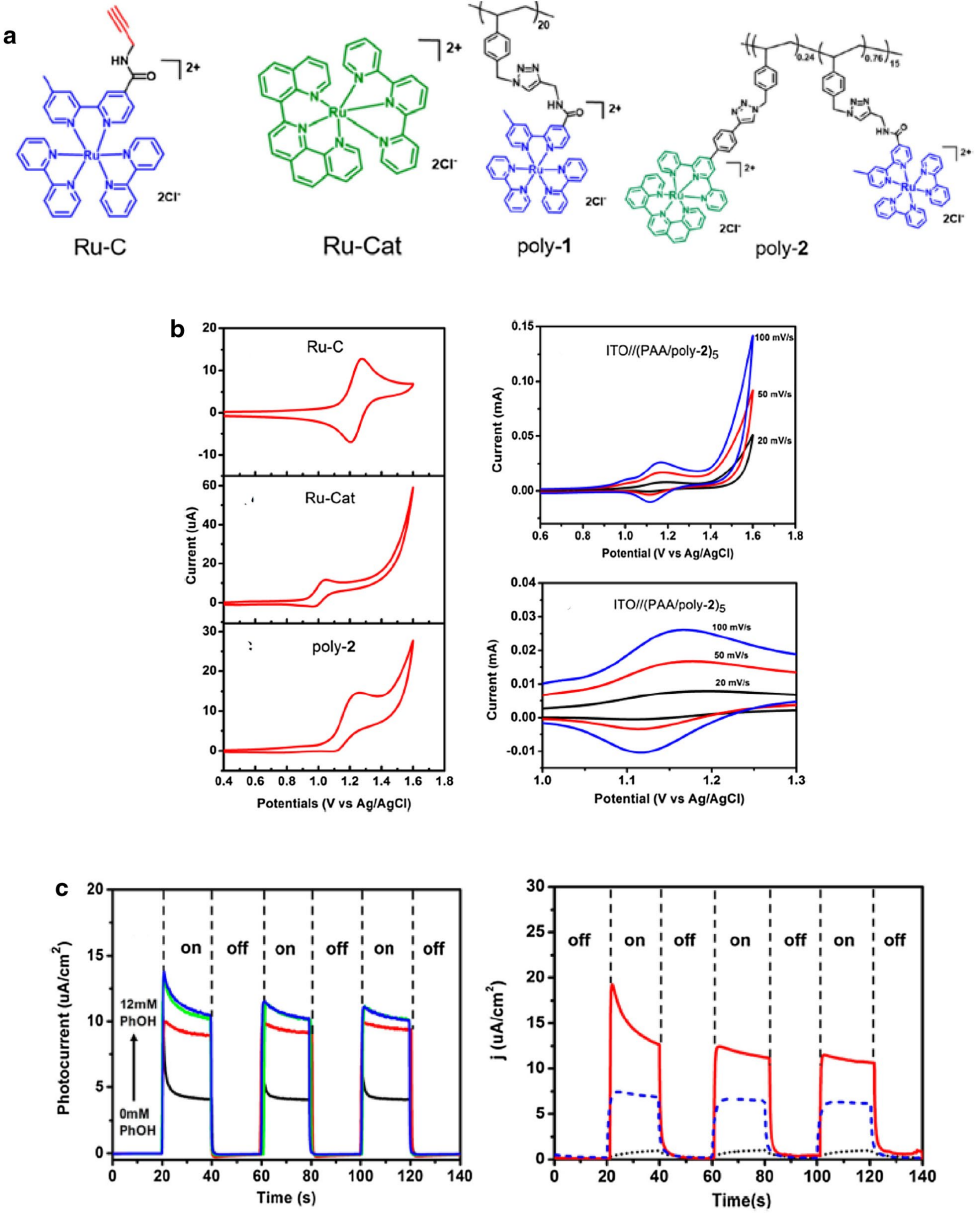
**a** Structures of Ru–C, Ru-Cat, poly-1, and poly-2. **b** Cyclic voltammograms of the indicated species in solution (left) and of the LbL films on an ITO electrode (right). **c** Photocurrent activity of FTO//nanoTiO_2_//(PAA/poly-2)_10_ with increasing concentrations of phenol in solution (left) and of FTO//(SnO_2_/TiO_2_)//(PAA/poly-2)_5_ (red), FTO//(SnO_2_/TiO_2_)//(PAA/poly-1)_5_ (blue, dashed), and FTO//(SnO_2_/TiO_2_) (black, dotted) in 0.1 M phosphate buffer at pH 7 (Reproduced with permission from Ref. [27]. Copyright 2017 American Chemical Society)

Recently, we elaborated on the LbL method with the use of a covalently linked Ru chromophore–catalyst polymeric assembly [27]. In contrast with the previous LbL study which involved the surface deposition of individual cationically charged **Ru**-**C** complexes, in this study the catalyst [Ru(trpy)(phenq)]^2+^ (**Ru**-**Cat**; troy = 2,2′:6,2″-terpyridine; phenq = 2-(quinol-8′-yl)-1,10-phenanthroline) was incorporated onto the same polymer backbone as the chromophore, and then deposited onto a mesoporous substrate via the LbL method. This approach enables the control of the chromophore-to-catalyst ratio within the polymer assembly. The chemical structure of a representative polymer, **poly-2**, is shown in Fig. 8. The **Ru**-**Cat** water oxidation catalyst and **Ru**-**C** chromophore moieties were attached to a polystyrene backbone via an azide-alkyne Huisgen cycloaddition reaction, with the relative ratio of the alkyne precursors of the chromophore and catalyst added to the click reaction feed dictating the ratio of each in the final polymer construct. Both ^1^H NMR and the comparison of the absorbance spectrum of the polymer with simulated component spectra of the **Ru**-**C** and **Ru**-**Cat** complexes verified the incorporation of each in the desired ratio into the final polymer strand.

Electrochemical analysis of **poly**-**2** showed behavior characteristic of both the **Ru**-**C** and **Ru**-**Cat** subunits (Fig. 8b). While the **poly**-**2** shows an anodic wave for the Ru^III/II^ couple of the **Ru**-**C** component at 1.2 V vs NHE and a catalytic wave at E_app_ > 1.4 V vs NHE characteristic of Ru-Cat, the oxidation of the **Ru**-**C** is irreversible in the polymer. This was taken as evidence for hole transfer from **Ru**-**C** ^**III**^ to **Ru**-**Cat** within the polymer structure. FTO//nanoTiO_2_ photoanodes with 10 LbL layers of PAA/poly-2 were active for the light driven oxidation of phenol or benzyl alcohol, with the photocurrent response increasing with an increase in concentration of the organic substrate (Fig. 8c). Light driven water oxidation was investigated with 5 LbL layer thick polyacrylic acid (PAA)/poly-2 on FTO//(SnO_2_/TiO_2_) core/shell electrodes (FTO//(SnO_2_/TiO_2_)//(PAA/poly-_2_)_5_) which gave a twofold enhanced photocurrent response compared to FTO//(SnO_2_/TiO_2_)//(PAA/poly-1)_5_ (poly-1 containing only **Ru**-**C** with no **Ru**-**Cat** moieties present in the polymer). This was taken as likely evidence for light driven water oxidation though the generation of O_2_ was not quantified.

## Summary

In this review, we summarize a series of polymeric assemblies consisting of multiple Ru(II) polypyridyl complexes prepared by using several polymerization methods including living anionic polymerization, RAFT, ATRP and NMP, followed by postreaction such as amidation reaction and click chemistry for the attachment of Ru(II) polypyridyl complexes to the polymer backbone. We describe the structure and dynamics of these polymer assemblies on mesoporous structure semiconductor films. Polymeric chromophore–catalyst assembly specifically containing chromophore units and an oxidation catalyst was developed to demonstrate its use in light-driven water oxidation at a photoanode-solution interface for a DSPEC application.
